# Occult coronary microvascular dysfunction and ischemic heart disease in patients with diabetes and heart failure

**DOI:** 10.1016/j.jocmr.2024.101073

**Published:** 2024-08-02

**Authors:** Noor Sharrack, Louise A.E. Brown, Jonathan Farley, Ali Wahab, Nicholas Jex, Sharmaine Thirunavukarasu, Amrit Chowdhary, Miroslawa Gorecka, Wasim Javed, Hui Xue, Eylem Levelt, Erica Dall’Armellina, Peter Kellman, Pankaj Garg, John P. Greenwood, Sven Plein, Peter P. Swoboda

**Affiliations:** a Leeds Institute of Cardiovascular and Metabolic Medicine, University of Leeds, Leeds, UK; b National Heart, Lung and Blood Institute, Bethesda, Maryland, USA; c Cardiovascular and Metabolic Medicine Group, Norwich Medical School, University of East Anglia, Norwich, UK

**Keywords:** Cardiovascular magnetic resonance (CMR), Coronary microvascular dysfunction (CMD), Diabetes mellitus (DM), Heart failure (HF), Myocardial blood flow (MBF), Myocardial perfusion reserve (MPR)

## Abstract

**Background:**

Patients with diabetes mellitus (DM) and heart failure (HF) have worse outcomes than normoglycemic HF patients. Cardiovascular magnetic resonance (CMR) can identify ischemic heart disease (IHD) and quantify coronary microvascular dysfunction (CMD) using myocardial perfusion reserve (MPR). We aimed to quantify the extent of silent IHD and CMD in patients with DM presenting with HF.

**Methods:**

Prospectively recruited outpatients undergoing assessment into the etiology of HF underwent in-line quantitative perfusion CMR for calculation of stress and rest myocardial blood flow (MBF) and MPR. Exclusions included angina or history of IHD. Patients were followed up (median 3.0 years) for major adverse cardiovascular events (MACE).

**Results:**

Final analysis included 343 patients (176 normoglycemic, 84 with pre-diabetes, and 83 with DM). Prevalence of silent IHD was highest in DM 31% ( 26/83), then pre-diabetes 20% (17/84) then normoglycemia 17%, ( 30/176). Stress MBF was lowest in DM (1.53 ± 0.52), then pre-diabetes (1.59 ± 0.54) then normoglycemia (1.83 ± 0.62). MPR was lowest in DM (2.37 ± 0.85) then pre-diabetes (2.41 ± 0.88) then normoglycemia (2.61 ± 0.90). During follow-up, 45 patients experienced at least one MACE. On univariate Cox regression analysis, MPR and presence of silent IHD were both associated with MACE. However, after correction for HbA1c, age, and left ventricular ejection fraction, the associations were no longer significant.

**Conclusion:**

Patients with DM and HF had higher prevalence of silent IHD, more evidence of CMD, and worse cardiovascular outcomes than their non-diabetic counterparts. These findings highlight the potential value of CMR for the assessment of silent IHD and CMD in patients with DM presenting with HF.

## Graphical abstract

**Figure:**
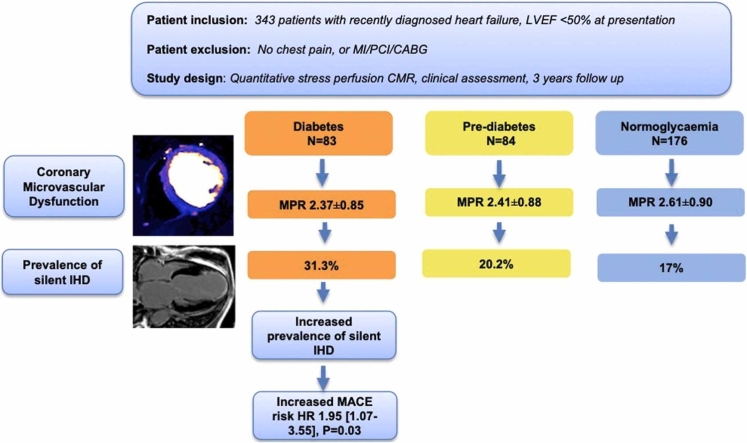

## 1 Introduction

Diabetes mellitus (DM) is associated with an increased risk of coronary artery disease (CAD), silent myocardial infarction (MI), ischemic cardiomyopathy, and heart failure (HF) [1], [2], [3]. HF is often the first manifestation of cardiovascular disease in patients with type 2 DM [2]. DM has been identified as an independent risk factor for the development of HF and prognosis in HF has been shown to be worse in those with DM [4], [5], [6], [7].

Silent MI is a relatively common finding in asymptomatic patients with DM and can be identified in as many as 28% of patients by cardiovascular magnetic resonance (CMR) [8], [9], [10], [11]. It is also seen in patients presenting with presumed dilated cardiomyopathy, even in those with unobstructed coronary arteries on invasive angiography [12]. The mechanisms leading to excess risk and worse cardiovascular outcomes in patients with HF and dysglycemia are not known [7]. There is limited evidence suggesting that the excess risk in patients with HF and dysglycemia relates to silent ischemic heart disease (IHD), but in the absence of coronary disease, patients with DM can still have a distinct HF phenotype often called “diabetic cardiomyopathy” [4]. The exact mechanisms underlying the development of this condition are unknown with a proposed mechanism of fibrosis and myocardial dysfunction progressing to systolic impairment [13]. Multiple studies have shown that coronary microvascular dysfunction (CMD) can be identified in patients with dysglycemia but its role in the etiology and adverse prognosis in patients with HF with dysglycemia is unknown [14], [15], [16]. Both stress myocardial blood flow (MBF) and the ratio of stress to rest MBF, termed myocardial perfusion reserve (MPR), serve as markers of CMD and can be measured using quantitative myocardial perfusion CMR, along with the assessment of both regional ischemia suggestive of flow-limiting CAD and previous MI.

We hypothesized that patients with dysglycemia presenting with a new diagnosis of HF have an increased prevalence of occult CAD and impaired coronary microvascular function. We aimed to investigate if either of these factors is associated with major adverse cardiovascular events (MACE) and whether the prognostic value of CMR findings differs between patients with HF with dysglycemia and normoglycemia.

## 2 Methods

### 2.1 Study population

In this prospective clinical study, 351 patients with newly diagnosed HF (with signs and symptoms of HF and a left ventricular ejection fraction (LVEF) <50% on referral echocardiogram within the last 12 months) who had been referred for a CMR scan to investigate etiology were recruited between February 2018 and January 2020 [17]. An LVEF <50% was chosen as a cutoff to capture patients with HF and reduced ejection fraction. Patients were excluded if they had a known history of CAD (coronary stenosis >70% on angiography, known MI, previous percutaneous coronary intervention, or coronary artery bypass grafting) or symptoms of angina. Other exclusion criteria included hypertrophic cardiomyopathy, amyloidosis, congenital heart disease, suspected acute pathology, such as myocarditis, advanced renal failure, or any contraindication to CMR or gadolinium-based contrast agents.

The primary outcome was a MACE, defined as the composite of cardiovascular death, non-fatal MI, stroke, and hospitalization due to HF or ventricular arrhythmia. Outcomes were captured by an annual review of National Health Service (NHS) medical records and death certificates by two clinical members of the team who both had to agree on a clinical event. Non-fatal MI included only spontaneous MI. Ventricular arrhythmia was defined as sustained ventricular tachycardia or ventricular fibrillation.

### 2.2 Patient characteristics

Patients underwent a clinical assessment on the day of their CMR appointment, including medical history, New York Heart Association (NYHA) functional class, risk factors, and current medications. Hematocrit (Hct), HbA1c, and B-type natriuretic peptide (BNP) were measured from a blood sample taken at the time of the CMR scan.

Patients were subsequently divided into normoglycemia (HbA1c <42 mmol/mol) and dysglycemia (HbA1c >42 mmol/mol), with this group being further divided into pre-diabetes (HbA1c 42–47 mmol/mol) and DM (pre-existing diagnosis of type 1 or 2 DM or HbA1c >47 mmol/mol) [1]. Silent IHD was defined as either inducible ischemia or MI on late gadolinium-enhanced (LGE) imaging.

### 2.3 Study protocol

All CMR studies were undertaken on a 3T system (Siemens Magnetom Prisma, Erlangen, Germany). Participants were instructed to abstain from caffeine for 24 h before the study. The protocol consisted of cine imaging, native and post contrast T1 mapping, stress and rest perfusion, and LGE. When it was unclear if the enhancement seen on bright blood LGE was ischemic, a dark blood LGE stack was also acquired for further clarification.

For stress perfusion imaging, adenosine was infused for a minimum of 3 min, at a rate of 140 µg/kg/min and increased up to a maximum of 210 µg/kg/min if there was insufficient hemodynamic response (heart rate increase less than 10 bpm or systolic blood pressure change less than 10 mmHg) or there was no symptomatic response, in line with standard clinical practice guidance [18]. Adequate stress was confirmed by perfusion color maps and splenic switch-off. Images were acquired during free breathing over 90 dynamics to allow for reduced blood transit times due to impaired ventricular function. A minimum 10-min interval was kept between stress and subsequent rest perfusion acquisitions.

Blood pressure and heart rate were recorded during adenosine infusion. For each perfusion acquisition, an intravenous bolus of 0.05 mmol/kg gadobutrol (Gadovist, Bayer Healthcare, Leverkusen, Germany) was administered at 5 mL/s followed by a 20 mL saline flush using an automated injection pump (Medrad MRXperion Injection System, Bayer Heathcare, Berlin, Germany). Perfusion mapping was performed using the Gadgetron streaming software image reconstruction framework [19].

### 2.4 Image analysis

Measurement of cardiac volume parameters and the presence of LGE were assessed using cvi42 software (Circle Cardiovascular Imaging, Calgary, Canada). LGE was reported if enhancement was identified on two orthogonal planes or, where available, on both bright and dark blood LGE images. Ischemic LGE was defined as involving the subendocardium in a typical coronary distribution, while non-ischemic LGE did not involve the subendocardium. Inducible ischemia was defined as a visual perfusion defect affecting ≥1 segment present at stress, but not at rest or matching an infarct on LGE imaging, in a coronary distribution.

Cvi42 was used to mark endocardial and epicardial borders (excluding papillary muscles) of parametric mapping and perfusion images. Right ventricular insertion points were marked, and a 16-segment American Heart Association model was used. To minimize the partial volume effect, a 10% offset was applied to endocardial and epicardial borders. T1 times and MBF were measured for each of the 16 segments as per our previous paper [20]. In-line automatic reconstruction, processing, and measurement of MBF were performed within the Gadgetron software framework as previously described [19]. Where the left ventricular outflow tract was erroneously included in a perfusion image, or partial volume effect meant segments were too thin to contour, these segments were excluded from further analysis. To report global MBF, (rather than the effects of occult IHD or replacement fibrosis) segments with visible regional perfusion defect or LGE were also excluded from the analysis to remove the effects of occult IHD on MACE. T1 times and MBF values for all remaining segments were averaged to provide a global value.

MPR was calculated as stress MBF/rest MBF. Extracellular volume fraction (ECV) was calculated using the formula “myocardial ECV = (1 − Hct) × (ΔR1myocardium/ΔR1blood), where R1 = 1/T1.” T1 and ECV values were calculated for basal, mid, and apical slices and averaged to provide a global value. Segments with ischemia, fibrosis, or infarction were excluded from analysis.

### 2.5 Statistical analysis

Analysis was performed using SPSS 23 (IBM SPSS, Armonk, New York ). Normality of distribution was assessed using the Shapiro-Wilk test. Data are presented as mean (±standard deviation) or median and interquartile range (IQR) for continuous data, and frequency (percentage) for categorical data.

Comparison between groups was performed using independent samples T-test or Mann-Whitney U test depending on normality and chi-square test or Fisher’s exact test for categorical data. Correlations were assessed using Pearson r correlation or Spearman’s rank correlation coefficient. Statistical tests were two-tailed and p < 0.05 was considered significant.

Cumulative hazard curves were constructed according to the Kaplan-Meier method and compared dichotomous groups using the presence of dysglycemia and median values of MPR within the study population as cutoffs. Where more than one MACE occurred to a patient, the first event was taken as an endpoint.

To identify independent predictors of MACE, separate Cox proportional hazard regression analyses were performed for variables, including age, sex, body mass index (BMI), LVEF, right ventricular ejection fraction (RVEF), T1 values, stress MBF, HbA1c, MPR, hypertension, and hypercholesterolemia for all patients. Multivariable regression was undertaken to assess whether silent IHD, stress MBF, or MPR was still associated with MACE after correction for age, LVEF, and HbA1c.

Based on our previous work [21], a sample size >326 would be required to detect a difference in MPR of 0.5 (standard deviation 1.2) between HF patients with and without DM (estimated prevalence of DM 25%, power 10%, and significance 5%).

## 3 Results

A total of 351 patients were recruited, of these 8 were excluded from the final analysis (3 due to a diagnosis of cardiac amyloidosis, 4 had contra-indications to adenosine, and 1 due to arrhythmia precluding accurate quantitative analysis).

Of the 343 patients included in the final analysis, 176 were normoglycemic (mean HbA1c 37 ± 3.3) and 167 dysglycemic, further divided into 84 with pre-diabetes (mean HbA1c 44 ± 1.6) and 83 with DM (mean HbA1c 57.2 ± 18.3).

### 3.1 Patient characteristics

Patient characteristics are shown in Table 1. Patients with DM were oldest, followed by patients with pre-diabetes and then normoglycemic patients. Patients with DM had a higher proportion of symptomatic patients, defined using NYHA class, followed by patients with pre-diabetes and then patients with normoglycemia. The prevalence of hypertension, hypercholesterolemia, and cerebrovascular disease was also highest in the DM group, followed by patients with pre-diabetes and then normoglycemia. Use of loop diuretics was lowest in normoglycemic patients and highest in the group with DM. The DM group was also more likely to be taking an angiotensin receptor inhibitor (ACEI).

**Table 1:** Patient characteristics.

		Normoglycemia (176)	Pre-diabetes (84)	Diabetes (83^a^)	p value for trend
Age (years)		61 (53–70)	65 (54–72)	67 (55–73)^b^	0.017^c^
Male		111 (63.1)	56 (66.7)	53 (63.9)	0.850
BMI (kg/m^2^)		28.1 ± 4.7	28.2 ± 5.3	28.3 ± 5.7	0.964
NYHA	I	127 (72.2)	50 (59.5)	43 (51.8)^b^	0.019^c^
	II	44 (25.0)	30 (35.7)	34 (41.0)	
	III	5 (2.8)	4 (4.8)	6 (7.2)	
HbA1c (mmol/mol)		37 ± 3.3	44.0 ± 1.6^d^	57.2 ± 18.3^b^	<0.001^c^
HbA1c (%)		5.54 ± 2	6.18 ± 2^d^	7.38 ± 4^b^	<0.001^c^
NT-proBNP (ng/L)		491 (158–1191)	629 (243–1932)	1098 (367–2369)	0.015^c^
SOBOE		70 (39.8)	34 (40.5)	33 (39.8)	0.993
Orthopnoea		24 (13.6)	15 (17.9)	13 (15.7)	0.667
Peripheral edema		26 (14.8)	10 (11.9)	14 (16.9)	0.658
Hypertension		72 (40.9)	35 (41.7)	48 (57.8)^b^	0.029^c^
Hypercholesterolemia		35 (19.9)	19 (22.6)	31 (37.3)^b^	0.009^c^
Stroke		21 (11.9)	6 (7.1)	15 (18.1)	0.097
Atrial fibrillation		66 (37.5)	32 (38.1)	36 (43.4)	0.650
Antiplatelet		31 (17.6)	18 (21.4)	16 (19.3)	0.761
Beta-blocker		127 (72.2)	68 (81.0)	71 (85.5)^b^	0.038^c^
Statin		58 (33.0)	38 (45.2)^d^	47 (56.6)^b^	0.001^c^
ACEI/ARB		140 (79.5)	66 (78.6)	79 (95.2)^b^	0.003^c^
MRA		34 (19.3)	19 (22.6)	28 (33.7)^b^	0.038^c^
Loop diuretic		52 (28.5)	37 (44.0)^d^	51 (61.4)^b^	<0.001^c^
Anticoagulant		55 (31.3)	30 (35.7)	30 (36.1)	0.678

*NYHA* New York Heart Association, *SOBOE* shortness of breath on exertion, *ACEI* angiotensin-converting enzyme inhibitor, *ARB* angiotensin receptor blocker, *MRA* mineralocorticoid antagonist, *NT-proBNP* N-terminal pro B-type natriuretic peptide, *BMI* body mass index . Continuous variables are presented as mean+/- standard deviation or mean and inter-quartile range. Dichotomous variables are presented as number (%).

a 73 patients with T2DM, 10 patients with T1DM.

b p < 0.05 for diabetes vs normoglycemia.

c p value <0.05 is considered significant.

d p < 0.05 for normoglycemia vs pre-diabetes.

### 3.2 CMR assessment

**Fig. 1:**
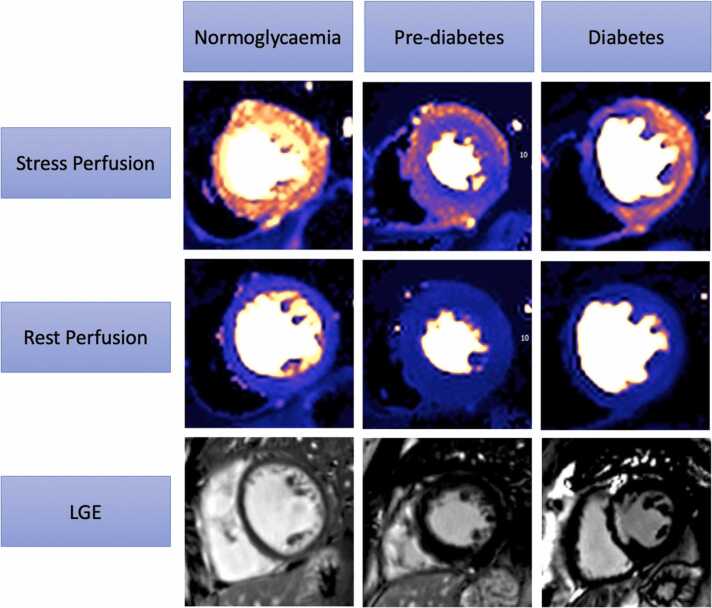
Stress and rest quantitative myocardial perfusion maps for three patients by glycemic status. In the normoglycemic patient, global stress perfusion is 3.0 mL/g/min, global resting perfusion is 1.0 mL/g/min, and MPR is 3. Late gadolinium enhancement (LGE) imaging demonstrates no enhancement. In the pre-diabetic patient, global stress perfusion is 1.85 mL/g/min, global resting perfusion is 0.87 mL/g/min, and MPR is 2.13. The polar maps are consistent with a diagnosis of coronary microvascular dysfunction. LGE demonstrates no enhancement. In the diabetic patient, global stress perfusion is 1.60 mL/g/min, global resting perfusion is 0.80 mL/g/min, and MPR is 2.0. The LGE demonstrates subendocardial enhancement of the mid-septum consistent with myocardial infarction in this territory. *MPR* myocardial perfusion reserve.

CMR data can be seen in Table 2. LVEF was lowest in the DM group, followed by the pre-diabetic group and highest in patients with normoglycemia. LV mass was highest in patients with DM and pre-diabetes compared to normoglycemia. No significant difference was seen in right ventricular parameters or left atrial size. The prevalence of silent IHD, defined as either inducible ischemia or MI on LGE, was highest in patients with DM compared to patients with pre-diabetes and normoglycemia. Stress MBF was lowest in DM (1.53 ± 0.52, p < 0.001 vs normoglycemia), then pre-diabetes (1.59 ± 0.54, p = 0.006 vs normoglycemia) then normoglycemia (1.83 ± 0.62). MPR was lowest in DM (2.37 ± 0.85, p = 0.04 vs normoglycemia), then pre-diabetes (2.41 ± 0.88, p = 0.76 vs normoglycemia) then normoglycemia (2.61 ± 0.90). Fig. 1 shows examples of quantitative perfusion maps and LGE in patients with diabetes, pre-diabetes, and normoglycemia.

**Table 2:** CMR assessment at baseline.

	Normoglycemia (176)	Pre-diabetes (84)	Diabetes (83)^a^	p value for trend
LVEF (%)	41.9 ± 11.9	39.6 ± 13.4	35.6 ± 12.5^b^	<0.001^c^
LVEDVi (mL/m^2^)	107.7 ± 33.1	112.7 ± 39.4	112.8 ± 37	0.424
LVMi (g/m^2^)	65.1 ± 18.4^d^	71.3 ± 18.7^d^	69.4 ± 19.5	0.029^c^
RVEDVi (mL/m^2^)	75.2 ± 19.6	78.3 ± 23.0	77.6 ± 24.3	0.478
RVEF (%)	50.3 ± 11.7	48.9 ± 13.9	47.9 ± 13.6	0.337
LAVi (mL/m^2^)	78.4 ± 35.4	84.6 ± 42.8	77.5 ± 36.8	0.393
Ischemia	10 (5.7)	4 (4.8)	9 (10.8)	0.215
Ischemic LGE	29 (16.5)	15 (17.9)	23 (27.7)^b^	0.094
Non-ischemic LGE	52 (30)	27 (32)	28 (34)	0.776
Ischemic heart disease	30 (17.0)	17 (20.2)	26 (31.3)^b^	0.030^c^
T1 (ms)	1316.5 ± 39.6	1331.0 ± 39.9^d^	1335.1 ± 45.2^b^	<0.001^c^
ECV (%)	25.1 ± 3.1	25.7 ± 2.9	25.8 ± 2.9	0.135
Stress MBF (mL/g/min)	1.83 ± 0.6	1.59 ± 0.5^d^	1.53 ± 0.5^b^	<0.001^c^
Stress systolic blood pressure (mmHg)	126.1 ± 20.0	127.2 ± 22.2	121.1 ± 20.6	0.07
Stress heart rate (bpm)	86.4 ± 16.5	81.2 ± 19.3	83.5 ± 16.0	0.41
Stress rate pressure product (bpm × mmHg)	10,947.3 ± 2694.2	10,274.8 ± 3004.1	10,096.2 ± 2636.0	0.70
Resting MBF (mL/g/min)	0.73 ± 0.2	0.69 ± 0.2	0.70 ± 0.3	0.305
Resting systolic blood pressure (mmHg)	126.1 ± 19.4	126.3 ± 21.1	121.0 ± 19.2	0.10
Resting heart rate (bpm)	71.6 ± 14.7	72.4 ± 18.3	71.2 ± 14.5	0.65
Resting rate pressure product (bpm × mmHg)	8962.6 ± 2082.2	9132.2 ± 2791.9	8667.0 ± 2426.8	0.26
MPR	2.61 ± 0.9	2.41 ± 0.9	2.37 ± 0.9^b^	0.064

*LVEF* left ventricular ejection fraction, *LVEDVi* indexed left ventricular end-diastolic volume, *LVMi* indexed left ventricular mass, *RVEDVi* indexed right ventricular end-diastolic volume, *RVEF* right ventricular ejection fraction, *LAVi* indexed left atrium volume, *LGE* late gadolinium enhancement, *ECV* extracellular volume fraction, *MBF* myocardial blood flow, *MPR* myocardial perfusion reserve, *bpm* beats per minute. Continuous variables are presented as mean+/- standard deviation or mean and inter-quartile range. Dichotomous variables are presented as number (%).

a 73 patients with T2DM, 10 patients with T1DM.

b p < 0.05 for diabetes vs normoglycemia.

c p value is significant at <0.05 level.

d p < 0.05 for normoglycemia vs pre-diabetes.

**Fig. 2:**
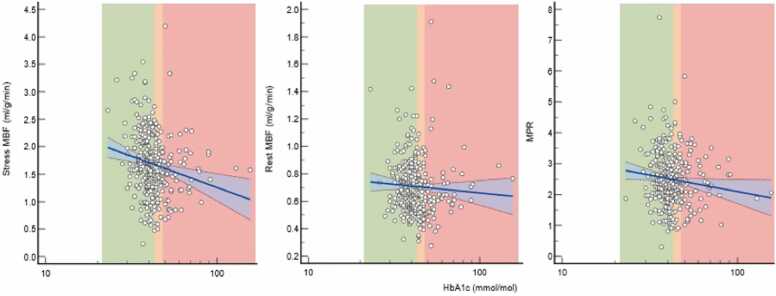
Associations between log-transformed HbA1c and stress MBF (left, R = −0.241, p < 0.001), rest MBF (middle, R = −0.140, p = 0.290) and MPR (right, R = −0.122, p = 0.039) with 95% confidence intervals shown in blue. The HbA1c thresholds for normoglycemia (green), pre-diabetes (orange), and diabetes (red) are shown. *MBF* myocardial blood flow, *MPR* myocardial perfusion reserve.

Significant associations were seen between glycemic control, measured as HbA1c and both stress MBF and MPR, but not resting MBF (Fig. 2).

Native T1 and ECV were highest in patients with DM, intermediate in patients with pre-diabetes, and lowest in patients with normoglycemia although only the differences in native T1 were statistically significant between groups. No significant difference was seen between the pre-diabetes and DM groups.

### 3.3 Outcomes

**Fig. 3:**
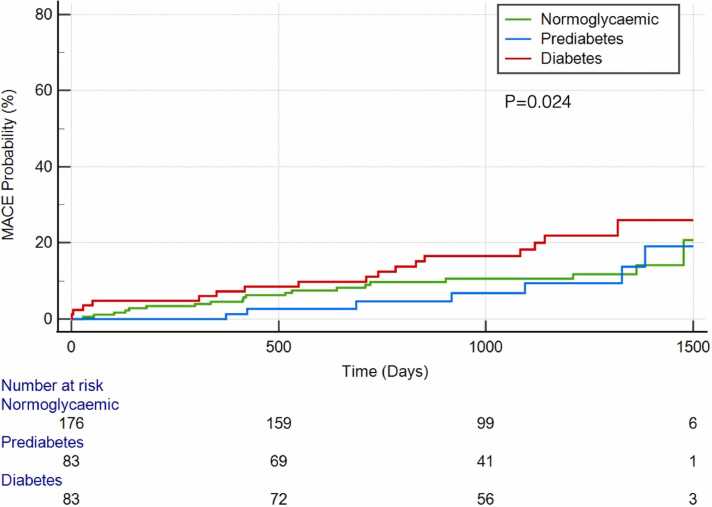
Kaplan-Meier survival curve by glycemic status. *MACE* major adverse cardiovascular events.

MACE data were available over a median follow-up period of 3 years (IQR 1.7–3.7 years). During this time, 45 patients suffered at least one MACE (Table 3), including 31 hospitalizations due to HF (9%), 9 strokes (2.6%), 13 cardiovascular deaths (3.8%), 4 acute coronary syndromes (ACS) (1.2%), and 4 episodes of ventricular arrhythmia (1.2%). Number of incident MACE events (all-cause death, HF admission, cardiovascular death, stroke, ACS and ventricular arrhythmia) are shown in Table 3. Median time to first MACE was 288 days (IQR 55–419). On univariate Cox regression analysis of all patients (Table 4), higher HbA1c and presence of DM (hazard ratio [HR] 1.95 [1.07–3.55], p = 0.03) were both significantly associated with increased risk of MACE (Table 4). After correction for age, HbA1c and LVEF neither silent IHD (HR 1.33 [0.50–3.55], p = 0.57), stress MBF (HR 0.55 [0.20–1.53,], p = 0.25), nor MPR (HR 1.10 [0.57–2.12], p = 0.77) had a significant association with MACE (Table 5). Kaplan-Meier event hazard curves, divided by glycemic status, are seen in Fig. 3.

**Table 3:** MACE events by glycemic status.

MACE event	Normoglycemia (176)	Pre-diabetes (84)	Diabetes (83)
Follow-up time (days)	1008 ± 385	1046 ± 384	921 ± 406
Total MACE	24 (14%)	8 (10%)	29 (35%)
Events per year	5.1	3.5	13.9
All-cause death	16 (9.1%)	9 (11%)	13 (15.7%)
HF admission	13 (7.4%)	5 (6%)	13 (15.7%)
CV death	4 (2.3%)	1 (1.2%)	8 (9.6%)
Stroke	3 (1.7%)	1 (1.2%)	5 (6%)
Non-fatal MI	2 (1.1%)	0	2 (2.4%)
VT/VF	2 (1.1%)	1 (1.2%)	1 (1.2%)

*MACE* major adverse cardiovascular events*, HF* heart failure, *CV* cardiovascular, *MI* myocardial infarction, *VT/VF* ventricular tachycardia/ventricular fibrillation*.* Continuous variables are presented as mean+/- standard deviation or mean and inter-quartile range. Dichotomous variables are presented as number (%).

**Table 4:** Univariate Cox regression analysis of association with MACE for all patients.

Covariate	Beta	SE	HR	95% CI of HR	p value
Age	0.044	0.014	1.045	1.016–1.074	0.002^a^
Sex	−0.205	0.322	0.815	0.433–1.532	0.525
BMI	−0.017	0.030	0.983	0.927–1.042	0.568
LVEF	−0.047	0.013	0.954	0.931–0.978	<0.001^a^
RVEF	−0.045	0.012	0.956	0.934–0.978	<0.001^a^
HbA1c	0.023	0.008	1.023	1.008–1.038	0.003^a^
Diabetes	0.668	0.305	1.950	1.072–3.547	0.029^a^
Pre-diabetes vs normoglycemia	0.221	0.440	1.248	0.527–2.953	0.615
T1	0.014	0.004	1.014	1.007–1.021	<0.001^a^
ECV	0.162	0.044	1.176	1.078–1.283	<0.001^a^
Stress MBF	0.492	0.276	0.611	0.356–1.049	0.074
Resting MBF	1.219	0.561	3.383	1.127–10.154	0.030^a^
MPR	−0.607	0.200	0.545	0.368–0.807	0.002^a^
IHD	0.886	0.308	2.425	1.327–4.433	0.004^a^
NI LGE	0.071	0.312	1.074	0.582–1.980	0.820
HTN	0.180	0.384	0.640	0.563–2.542	0.640
Hypercholesterolemia	0.023	0.445	1.023	0.428–2.445	0.959

*BMI* body mass index, *LVEF* left ventricular ejection fraction, *RVEF* right ventricular ejection fraction, *ECV* extracellular volume fraction, *MBF* myocardial blood flow, *MPR* myocardial perfusion reserve, *IHD* ischemic heart disease, *LGE* late gadolinium enhanced, *NI LGE* non-ischemic late gadolinium enhancement, *CI* confidence interval, *MACE* major adverse cardiovascular events.

Univariate Cox regression analysis of association with MACE. Increasing age, HbA1c, rest MBF, T1, ECV, and IHD were associated with increasing risk. Increasing LVEF and RVEF were associated with reduced risk, *HR* hazard ratio, *HTN* hypertension, *SE* standard error. Continuous variables are presented as mean+/- standard deviation or mean and inter-quartile range. Dichotomous variables are presented as number (%).

a p value considered significant at 0.05 level.

**Table 5:** Multivariate analysis of association of MACE for all patients.

Covariate	Beta	SE	HR	95% CI of HR	p value
Silent IHD	0.28	0.50	1.33	0.50–3.55	0.57
Stress MBF	−0.60	0.53	0.55	0.20–1.53	0.25
MPR	0.10 6	0.334	1.10	0.57–2.12	0.77

*MACE* major adverse cardiovascular events*, CI* confidence interval*, IHD* ischemic heart disease*, MBF* myocardial blood flow*, MPR* myocardial perfusion reserve*.* All covariates were corrected for age, HbA1C and LVEF." *HbA1C* glycated haemoglobin, *LVEF* left ventricular ejection fraction, *HR* hazard ratio. Continuous variables are presented as mean+/- standard deviation or mean and inter-quartile range. Dichotomous variables are presented as number (%)

## 4 Discussion

In this prospective study of 343 patients with newly diagnosed HF without symptoms or history of IHD, both the prevalence of silent IHD and extent of CMD (measured as stress MBF) were highest in patients with DM, intermediate in patients with pre-diabetes, and lowest in patients with normoglycemia. In keeping with previously published literature [2], [5], a diagnosis of DM was associated with adverse outcomes on follow-up [2], [5].

### 4.1 Silent IHD in diabetes

Within our cohort, we found an increased prevalence of silent IHD in patients with DM 31%, (26/83) and then patients with pre-diabetes 20%, (17/84) compared to 17%, (30/176) in the normoglycemic group. This is similar to the prevalence in the literature where previous studies have shown the incidence of silent MI in patients with DM to range between 17% and 28% [8], [10], [11]. Another study also examined patients with impaired fasting glucose and found an incidence of silent MI of 16%, suggesting this risk extends to patients with pre-diabetes [11]. The higher prevalence of silent IHD in patients with DM may be partly explained by cardiac autonomic neuropathy although the higher presence in patients with pre-diabetes, when cardiac autonomic neuropathy is unlikely to be present, suggests additional mechanisms [22]. In our study at baseline, there was only modest use of statins (57%) and antiplatelets (19%). There is therefore a potential role for testing for silent IHD and subsequent treatment with antiplatelets and high-intensity statins.

In our study, silent IHD was associated with adverse outcomes in patients with DM which is consistent with previous studies, such as ICELAND MI [23]. This was a study of 936 participants, in which the MACE rate was higher in those with silent IHD. We have also shown that silent IHD was associated with adverse outcomes in the normoglycemic group (HR 2.4, p = 0.004), which is similar to the findings seen in ICELAND MI.

### 4.2 Coronary microvascular dysfunction

The coronary microcirculation has a fundamental role in the regulation of coronary blood flow in response to cardiac oxygen requirements. Impairment of this mechanism, defined as CMD, carries an increased risk of adverse cardiovascular clinical outcome. Both stress MBF and MPR serve as markers of CMD and have been validated against invasive measures of coronary physiology [24]. We found that in regions of LV myocardium without infarction, fibrosis, or ischemia, stress MBF and MPR were both lowest in patients with DM, intermediate in patients with pre-diabetes, and highest in patients with normoglycemia (Table 2). In patients with DM, both stress MBF and MPR were lower than in normoglycemic patients, whereas in patients with pre-diabetes only the stress MBF was significantly lower than in normoglycemic patients. This suggests that stress MBF may be more sensitive for detection of CMD in patients with DM.

Previous CMR and PET studies have identified a reduction in MPR in patients with DM without known HF, and impairment of MPR has been shown to be a prognostic marker in both CMR and PET studies [21], [25], [26].

The mechanisms by which dysglycemia leads to CMD and diabetic cardiomyopathy remain a subject of debate. One explanation relates to microvascular remodelling, capillary basement thickening, and microaneurysm formation causing vasoconstriction and lower coronary blood flow [27], [28]. This process is associated with downregulation of nitric oxide production in hyperglycemia [29]. Other mechanisms include the activation of protein kinase C, as well as the activation of the polyol pathway and cardiac autonomic neuropathy [28].

### 4.3 Associations between glycemic control, CMD, and outcomes

The high prevalence of DM in our HF cohort (24%) is in keeping with studies that show the prevalence of known DM in HF patients to range between 13% and 47% [30]. Across the whole cohort, we found significant correlations between HbA1c and both stress MBF and MPR (Fig. 2). We found significant associations between the presence of silent IHD and CMD (both stress MBF and MPR).

In diabetic cardiomyopathy, higher HbA1c has been shown to be associated with worse outcomes, although this relationship is not linear, with poor outcomes seen in those with low HbA1c as well [31]. While recent studies using new agents, such as sodium glucose cotransporter-2 inhibitors, have shown improved HF outcomes, the effects demonstrated have not been explained by improved glycemic control and other studies have shown no improvement in HF outcomes with better glycemic control [32], [33]. Although we have shown significant associations between both CMD and glycemic control, it remains to be proven whether CMD has a mechanistic role in diabetic cardiomyopathy or is a non-causative correlation.

## 5 Limitations

This was a single-center study and findings need to be replicated in randomized controlled multicenter studies. Since patients recruited to this study were referred for CMR as part of routine clinical practice, referral bias may have excluded frailer patients, or patients with more severe HF, who were not suitable to undergo CMR. This may in part reflect why our reported MACE rate was lower than contemporary HF clinical trials. Most of our patients had few symptoms and were classed as NYHA I which may reflect the fact that they had already been started on optimal medical treatment before their CMR scan. Furthermore, although we excluded CMR segments with regional perfusion defects and infarction from global perfusion measurements, since patients did not routinely undergo invasive coronary angiography, we cannot completely exclude the presence of CAD as reduced global perfusion may also be caused by diffuse epicardial disease. In this study, we have combined patients with both type 1 and type 2 DM who may have differing coronary microvascular responses to hyperglycemia. Furthermore, patients in the study with diabetes were overall significantly older, had higher NT-pro-BNP levels, lower LVEF, a higher prevalence of hypertension, and a higher percentage of previous cardiovascular events. The increased MACE rate is therefore not unexpected and raises the question of the extent to which the CMR findings represent independent risk parameters.

## 6 Conclusions

Patients with DM and HF, even in the absence of symptoms, had higher prevalence of silent IHD, more evidence of CMD, and worse cardiovascular outcomes than their non-diabetic counterparts. These findings highlight the potential value of CMR for assessment of silent IHD and CMD in patients with DM presenting with HF. Future studies are needed to establish whether either silent IHD or CMD could be a therapeutic target.

## Funding

This research was funded by 10.13039/501100000274British Heart Foundation (RG/16/1/32092). S.P. is supported by a British Heart Foundation Chair (CH/16/2/32089). E.L. acknowledges support from the 10.13039/100004440Wellcome Trust (221690/Z/20/Z). This research is supported by the 10.13039/501100000272National Institute for Health Research (NIHR) through the Local Clinical Research Networks and the Leeds Clinical Research Facility.

## Author contributions

Study concepts/study design or data acquisition or data analysis/interpretation: N.S., L.A.E.B., J.F., A.W., N.J., S.T., A.C., M.G., W.J., H.X., E.L., E.D., P.K., S.P., P.P.S.; manuscript drafting or manuscript revision for important intellectual content: N.S., L.A.E.B., S.P., P.P.S.; approval of final version of submitted manuscript: all authors; agrees to ensure any questions related to the work are appropriately resolved: all authors; statistical analysis: N.S., L.A.E.B., P.P.S., P.G.; and manuscript editing: N.S., L.A.E.B., S.P., P.P.S. PPS is the guarantor of this work and, as such, had full access to all the data in the study and takes responsibility for the integrity of the data and the accuracy of the data analysis.

## Ethics approval and consent

The CMR registry was approved by the NHS research ethics committee (17/YH/0300) and the patients provided written informed consent for their inclusion. This study complies with the Declaration of Helsinki.

## Consent for publication

Consent for publication was obtained from all authors.

## Declaration of competing interests

The authors declare that they have no known competing financial interests or personal relationships that could have appeared to influence the work reported in this paper.

## Data Availability

The datasets used and analyzed during the current study are available from the corresponding author on reasonable request.
